# COVID-19 Vaccine Hesitancy and Emerging Variants: Evidence from Six Countries

**DOI:** 10.3390/bs11110148

**Published:** 2021-10-28

**Authors:** Sherry Mangla, Fatima Tuz Zohra Makkia, Ashok Kumar Pathak, Renee Robinson, Nargis Sultana, Kranthi Swaroop Koonisetty, Ajlina Karamehic-Muratovic, Uyen-Sa D.T. Nguyen, Alfonso J. Rodriguez-Morales, Jorge A. Sanchez-Duque, Patrick T. Zamba, Nasrin Aghamohammadi, Fong CS, Ubydul Haque

**Affiliations:** 1 International Institute for Population Sciences, Mumbai 400088, Maharashtra, India; sherry.mangla97@gmail.com; 2 Department of Epidemiology, Human Genetics and Environmental Sciences, University of Texas Health Science Center, 1200 Pressler Street, Houston, TX 77030, USA; fatima.zohra.makkia@gmail.com; 3 Department of Mathematics and Statistics, Central University of Punjab, Bathinda 151401, Punjab, India; ashokiitb09@gmail.com; 4 University of Alaska Anchorage/Idaho State University College of Pharmacy, 2533 Providence Drive, PSB 108B, Anchorage, AK 99508, USA; reneerobinson@isu.edu; 5 Office of Public Health Studies, Thompson School of Social Work and Public Health, University of Hawai’i at Mānoa, Honolulu, HI 96822, USA; nargis@hawaii.edu; 6 Department of Biostatistics and Epidemiology, University of North Texas Health Science Center, Fort Worth, TX 76177, USA; kranthiswaroop93@gmail.com (K.S.K.); Uyen-sa.Nguyen@unthsc.edu (U.-S.D.T.N.); patrickzamba@my.unthsc.edu (P.T.Z.); 7 Department of Sociology and Anthropology, St. Louis University, St. Louis, MO 63108, USA; ajlina.karamehicmuratovic@slu.edu; 8 Grupo de Investigación Biomedicina, Faculty of Medicine, Fundacion Universitaria Autonoma de las Américas, Pereira 660003, Colombia; alfonso.rodriguez@uam.edu.co or; 9 Department of Clinical Epidemiology and Biostatistics, Universidad Científica del Sur, Lima 15046, Peru; 10 Grupo de Investigación Salud, Familia y Sociedad, Department of Social Medicine and Family Health, Faculty of Health Sciences, Universidad del Cauca, Popayán 660003, Colombia; jorgesd@unicauca.edu.co; 11 Centre for Epidemiology and Evidence-Based Practice, Department of Social and Preventive Medicine, University of Malaya Kuala Lumpur, Kula Lumpur 50603, Malaysia; nasrin@ummc.edu.my; 12 Institute for Advanced Studies, University of Malaya, Kuala Lumpur 50603, Malaysia; fongcs92@gmail.com

**Keywords:** COVID-19 vaccination, SARS-CoV-2 vaccine, immunization programs, pandemics

## Abstract

As the world tries to cope with the devastating effects of the COVID-19 pandemic and emerging variants of the virus, COVID-19 vaccination has become an even more critical tool toward normalcy. The effectiveness of the vaccination program and specifically vaccine uptake and coverage, however, is a function of an individual’s knowledge and individual opinion about the disease and available vaccines. This study investigated the knowledge, attitudes, and resulting community practice(s) associated with the new COVID-19 variants and vaccines in Bangladesh, Colombia, India, Malaysia, Zimbabwe, and the USA. A cross-sectional web-based Knowledge, Attitudes, and Practices (KAP) survey was administered to respondents living in six different countries using a structured and multi-item questionnaire. Survey questions were translated into English, Spanish, and Malay to accommodate the local language in each country. Associations between KAP and a range of explanatory variables were assessed using univariate and multiple logistic regression. A total of 781 responses were included in the final analysis. The Knowledge score mean was 24 (out of 46), Attitude score 28.9 (out of 55), and Practice score 7.3 (out of 11). Almost 65% of the respondents reported being knowledgeable about COVID-19 variants and vaccination, 55% reported a positive attitude toward available COVID-19 vaccines, and 85% reported engaging in practices that supported COVID-19 vaccination. From the multiple logistic models, we found post-graduate education (AOR = 1.83, 95% CI: 1.23–2.74) and an age range 45–54 years (AOR = 5.81, 95% CI: 2.30–14.69) to be significantly associated with reported COVID-19 knowledge. In addition, positive Attitude scores were associated with respondents living in Zimbabwe (AOR = 4.49, 95% CI: 2.04–9.90) and positive Practice scores were found to be associated with people from India (AOR = 3.68, 95% CI: 1.15–11.74) and high school education (AOR = 2.16, 95% CI: 1.07–4.38). This study contributes to the identification of socio-demographic factors associated with poor knowledge, attitudes, and practices relating to COVID-19 variants and vaccines. It presents an opportunity for collaboration with diverse communities to address COVID-19 misinformation and common sources of vaccine hesitancy (i.e., knowledge, attitudes, and practices).

## 1. Background

Over 20-million deaths worldwide have been directly attributed to infectious diseases such as influenza, Severe Acute Respiratory Syndrome (SARS), H5N1 influenza (bird flu), and, most recently, severe acute respiratory syndrome coronavirus 2 (SARS-CoV-2), the virus responsible for coronavirus disease 2019 (COVID-19) and variants of interest (VOIs): Epsilon (B.1.427 and B.1.429); Zeta (P.2); Eta (B.1.525); Theta (P.3); Iota (B.1.526); Kappa (B.1.617.1); Lambda (C.37) and Mu (B.1.621); as well as variants of concern (VOCs), such as Alpha (B.1.1.7), Beta (B.1.351), Gamma (P.1), and Delta (B.1.617.2) [1,2,3]. Vaccination remains one of the most effective ways to reduce COVID-19-related mortality and morbidity, slowing viral transmission, lessening disease severity, and reducing the number of variants [4]. 

For as long as vaccines have been in existence, vaccine hesitancy has coexisted and hinders the success of immunization [5]. According to the WHO SAGE group, “vaccine hesitancy refers to a delay in acceptance or refusal of vaccination despite the availability of vaccination services.” [6,7]. Similar to the recent experiences with the COVID-19 vaccine hesitancy, there were misinformation, skeptics, and doubters of the effectiveness of smallpox and influenza vaccines worldwide. In an example from an Australian study, likely responses from the general public included “the situation is not serious enough” and “I am not at risk” [8]. Similar studies worldwide showed vaccine hesitancy that manifested from doubt on the efficacy of a vaccine, to lack of general knowledge about disease and effectiveness of vaccines, as well as misconceptions and false statements about vaccines [9]. A survey of the US population in 2009 showed that the greater the trust in the government, the more likely people will be vaccinated while those with the least confidence in government will be more likely to have vaccine hesitancy; thus, policymakers must build trust to have greater compliances in vaccine uptake [10].

Within less than 18 months of the first confirmed COVID-19 case, biopharmaceutical companies partnered with governmental entities and external researchers to develop three COVID-19 vaccines. Meanwhile, more than 200,000 COVID-19-related scientific articles were released [11]. However, despite the unprecedented speed of vaccine development and robust global mass vaccination efforts, vaccine acceptance and uptake have been sub-optimal. Ongoing research suggests that 70% or more of the United States of America (USA) population will need to receive the COVID-19 vaccine to achieve herd immunity. Unlike the recent widespread global acceptance of noted vaccines such as influenza, pneumococcal, and varicella and immunization programs, there has been less acceptance of the COVID-19 vaccines [12,13,14]. In the US, current and projected COVID-19 vaccine uptake is far lower than the 70% necessary [15,16,17] to achieve herd immunity. Moreover, the subsequent emergence of new SARS-CoV-2 variants threatens the progress made in limiting the spread of this virus [18]. 

Vaccine misinformation presents a significant but modifiable barrier to local, national, and international vaccination programs, and reduces vaccine acceptance and uptake. Waning public confidence in the COVID-19 vaccine and trust in the government, as well as in medical suppliers and the healthcare system, remains a significant concern. Many individuals worldwide now are choosing not to be vaccinated, citing expedited vaccine development timelines, concerns with technologies employed, and other external influencers (e.g., political, religious, etc.) as the reason for vaccine hesitancy and reduced vaccine confidence [19,20,21,22]. A climate of respect and trust between science (e.g., healthcare systems, providers, and drug manufacturers) and society (e.g., government and community) is essential for compliance with COVID-19 guidelines. Steps must be taken to support information transparency, vigilant fact-checking, and the cultivation of scientific knowledge [23]. This study investigated the knowledge, attitudes, and resulting community’s COVID-19 risk mitigation and management practice(s) in six countries including Bangladesh, Colombia, India, Malaysia, Zimbabwe, and the USA. 

## 2. Methodology 

The current study utilized a web-based cross-sectional survey of the Knowledge, Attitude, and Practices (KAP) model. The KAP study employs predefined formatted questions to solicit quantitative information from the respondent’s existing knowledge about COVID-19 variants, virulence, perceived knowledge about vaccines, religious beliefs, and the roles of media and its effects on vaccine uptake. KAP surveys reveal misconceptions or misunderstandings that may represent obstacles to the activities that we would like to implement, and potential barriers to practice change. The survey was initially prepared in the English language and then translated into Spanish and Bahasa Malaya (Malaysian National Language) to accommodate local languages and ensure responses from countries like Bangladesh, Colombia, India, Malaysia, and Zimbabwe. The translated questionnaire was pre-tested for validity and reliability in each country. The survey was shared via the authors’ networks using Google forms on social media platforms such as Facebook, Twitter, LinkedIn, WhatsApp groups, and Instagram, for two months (between June and July 2021). The survey was set to be taken only once from a mobile phone or computer, ensuring that no duplicate responses were recorded. Data from a total of 781 entries were received and analyzed.

Of note, the study sample was not a randomly selected sample from the aforementioned countries, as the study population consisted of many participants from the authors’ networks. As the experience of study participants may not necessarily reflect the experience of the general population in each country, there is potential for self-selection bias. Moreover, the collection of data using web-based instruments further restricted the sample to those with access to computers and the internet. Nevertheless, the sample included a large proportion of people between 15 and 34 years of age and people with less than a high-school education. 

### 2.1. Questionnaire 

The web-based KAP questionnaire had three sections (Supplementary Materials-appendix). In addition to Knowledge, Attitudes and Beliefs, and Practice, we included a section on Demographics to record the demographic characteristics of the study population. The respondents’ self-reported demographic data included age, sex, nationality, country, state of residence, level of education, employment status, and occupation. No personal identifiers were collected in the survey. The Knowledge section in the questionnaire had a total of 17 multiple-choice items, where the respondents were asked to identify the names of new COVID-19 variants, countries of origin for variants, symptoms, diagnostic tests, and the respondent’s source of information regarding the new variants. In a section on Vaccination Status, the respondents were asked about their status of vaccination, the name of the vaccine, side effects experienced due to the vaccine, and if they had breakthrough COVID-19 infection after getting vaccinated. In the Attitudes and Beliefs section, respondents’ perspectives on safety, effectiveness, and community involvement regarding the COVID-19 vaccine were assessed. Eleven statements were included in this section, and the respondents were asked to choose from the multiple options. A summary statistic of COVID-19 variants and vaccine knowledge is provided in Table S1a, and Table S1b respectively. These items in Attitude helped us gauge respondents’ vaccine hesitancy (Table S2). The Practice section had six questions in total. The respondents were asked about their change in practices and plans due to new variants of COVID-19 (Table S3). 

### 2.2. Statistical Analysis

Seventeen, eleven, and six items on the questionnaire were used to define the level of knowledge, attitude, and practice, respectively. For each item, a score was given, keeping in mind the expected responses. For a particular domain of KAP, some but not all questions have a score in the range from 0 to 10 (knowledge), 1 to 5 (attitude), or 0 to 2 (practice), depending upon the type of question and responses received. Thus, an individual’s maximum total KAP score could be 47, 55, and 11, respectively. More detailed information on the scoring system is provided in Table S4. The first part of the analysis determined the association between socio-demographic variables and the KAP scores. To do this, the KAP scores were converted into binary variables (good/poor). The definition of good/poor score was based on the overall median score in each KAP questionnaire. Respondents with a Knowledge score greater than 23.5 were categorized as having “good knowledge;” otherwise, the response was categorized as poor. For Attitude and Practice scores, the cut-off value was 27.5 and 5.5, respectively. In addition, we also examined the proportions of those who were concerned about side effects (i.e., if response for COVID-19 vaccines contains dangerous ingredients), those who did not believe in the vaccine (i.e., if the response does not have enough information about the COVID-19 vaccine), or those who did not believe its effectiveness to gauge vaccine hesitancy (i.e., if response for COVID-19 vaccines is not effective at preventing COVID-19 infection). Next, a comparison between univariate and multiple logistic regression was made. Here, we showed the effect of an independent variable when we control the other independent variables. Univariate logistic regression was run to explore the associations between different demographic variables and the KAP levels. We then assessed all socio-demographic variables (i.e., sex, age group, country, education, and employment) using multiple logistic models. The results of all logistic regression analyses are reported as unadjusted odds ratios (OR) for univariate logistic regression and adjusted odds ratio (AOR) for multiple logistic regression with 95% confidence intervals (95% CI) and *p* ≤ 0.05. Correlations between the respondent’s total knowledge, attitude, and practice were determined to assess the relationship among the KAP scores. Spearman’s rank correlation coefficient (r) and the corresponding *p*-values (≤0.05) were used because of the non-normality of the dataset. All the analyses were done using MS-Excel 2016 and Stata Version 16.0.

## 3. Results

### 3.1. Respondent Characteristics

**Figure 1:**
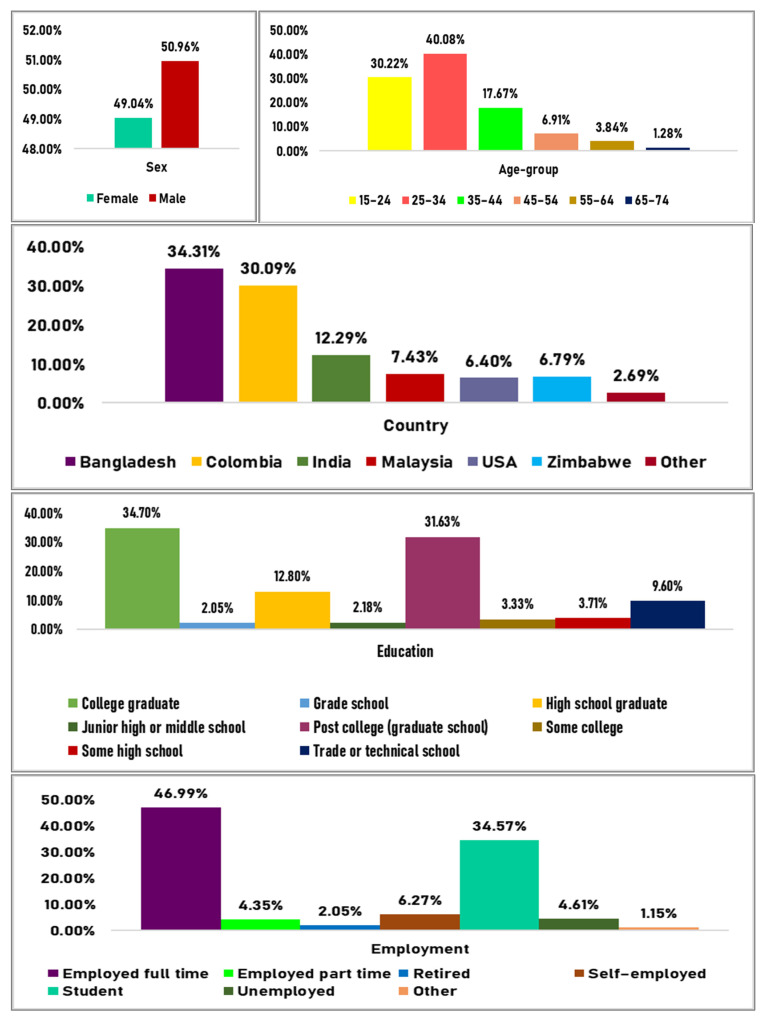
Sociodemographic characteristics of the study respondents.

Of the 781 respondents, 49.04% were female, 47% were full-time workers, 40% were aged 25–34 years, and approximately 1.28% were aged between 65–74 years. More than one-third of the respondents reported currently living in Bangladesh (34.3%) or Colombia (30%), followed by India (12.29%), Malaysia (7.43%), Zimbabwe (6.79%), and the USA (6.40%). Respondents in the study from countries other than these accounted for 2.69% of the total response givers. As many as 271 (34.7%) respondents reported being college graduates, 247 (31.63%) completed their post-graduation, and 16 (2.05%) completed elementary (grade) school. More detailed information on the socio-demographic characteristics of the study respondents can be seen in Figure 1.

### 3.2. Scores

**Figure 2:**
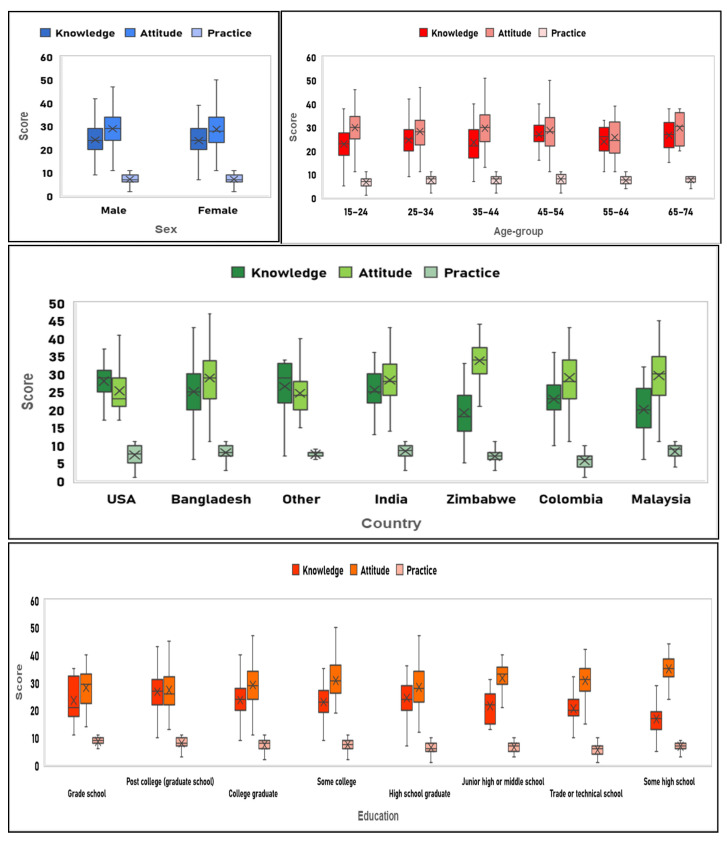

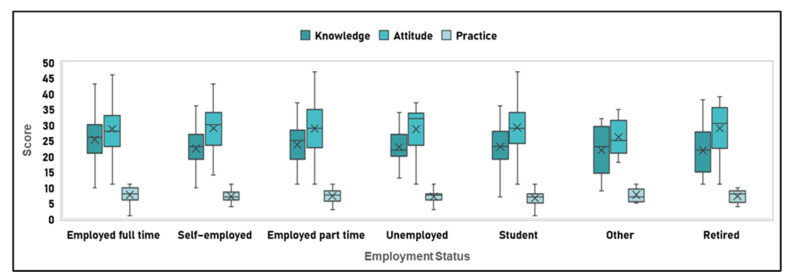
Box-plot of the Knowledge, Attitude, and Practice scores among different socio-demographic categories.

The overall mean knowledge, attitude, and practice score were 24 (out of 47), 28.9 (out of 55), and 7.3 (out of 11) respectively. Figure 2 displays a box plot of the KAP scores indicating the dispersion based on demographics. Respondents aged 45–54 had the highest mean Knowledge (27) and Practice (7.9) score, whereas the 15–24 and 65–74 age group respondents had a maximum Attitude score (29.8). The minimal variation in mean Knowledge, Attitude, and Practice scores was noted by gender. 

### 3.3. Relationship between Knowledge, Attitude, and Practice

**Figure 3:**
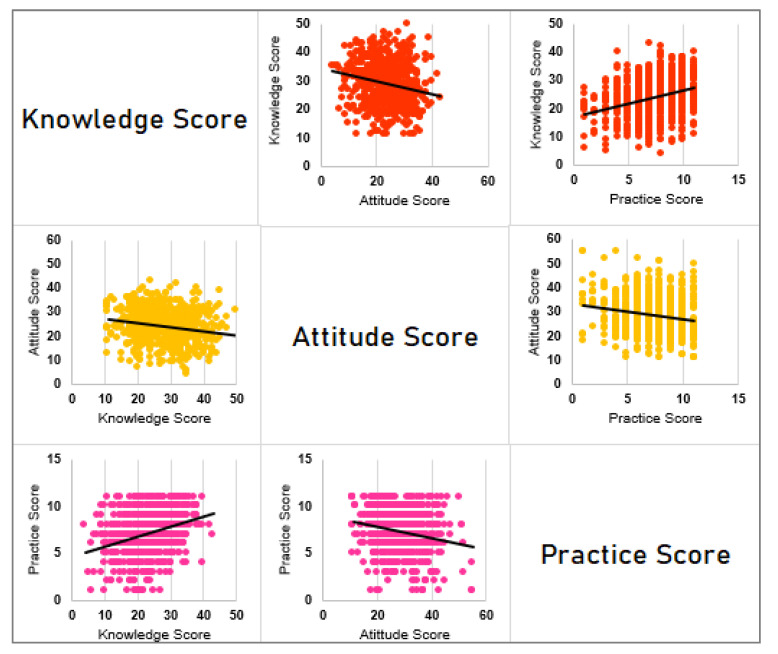
Scatter plot demonstrating the relationship between total knowledge, attitude, and practice score towards COVID-19 variants and vaccines.

Significantly weak and negative correlations between the Knowledge and Attitude scores (r= −0.23, *p* = 0.0001) and Attitude Practice scores (r= −0.18, *p* = 0.003) were noted. However, the maximum positive correlation was found between Knowledge and Practice scores (r = 0.32, *p* = 0.000006). The scatterplot matrix in Figure 3 shows the extent of the relationship between the three KAP scores.

### 3.4. Knowledge

**Figure 4:**
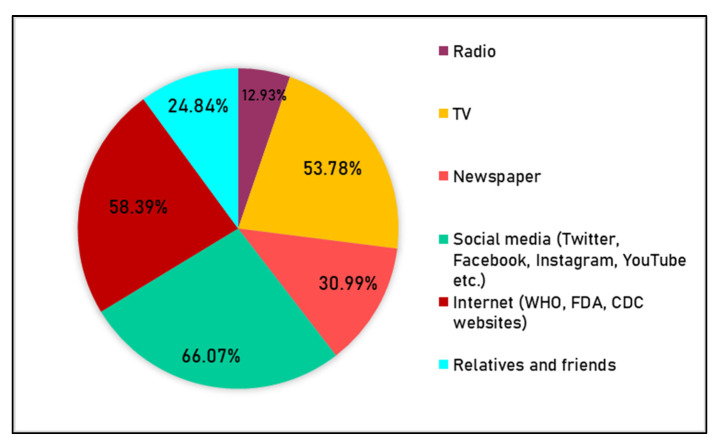
The popularity of different sources of information among the study respondents.

Of the 781 respondents, 41.1% claimed that they could identify the total number of new COVID-19 variants, with only 27 of these 321 people who correctly identified all-new variants, as shown in Table S1a. Approximately 80% of the respondents knew that the new variants were more contagious than the actual COVID-19 virus, 16% were unaware of the increased virulence, and nearly 57% of the study population believed new variants could be detected using available diagnostic tests. However, when asked about necessary measures required to control variant spread, the majority of respondents answered correctly, with only 3% reporting being not sure. Social media and the internet were the primary sources of information for most respondents—66% and 58%, respectively (Figure 4).

**Figure 5:**
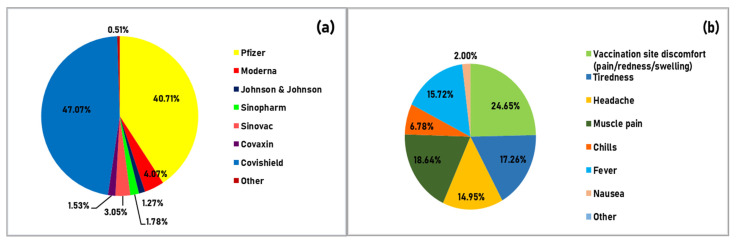
(**a**) Distribution of different vaccines taken by the study respondents (*n* = 393), (**b**) Common side-effects after the vaccination, experienced by the respondents (*n* = 393).

In terms of COVID-19 vaccine knowledge, only 393 respondents indicated that they had received at least one dose of vaccine and were eligible to complete the survey, as indicated in Table S1b. People who have taken the vaccine, irrespective of doses, the vaccine taken, side effects, etc., have been given the same score, i.e., 1, in comparison to those who did not take the vaccine, which indicates that those who received the vaccines were more knowledgeable about vaccination. A majority of the respondents completed both doses of their vaccine, with “Covishield” and “Pfizer” being the most common (see Figure 5a). Approximately 61% of the respondents who have been vaccinated experienced vaccine side effects, muscle pain being the most common side effect (see Figure 5b). However, 15 respondents who received both the doses and 55 respondents who received only one dose of vaccine reported a subsequent COVID-19 infection. Table S1a,b lists Knowledge questions, expected answers, and the percentage of the respondents who gave the expected answer.

In the univariate analysis, fifteen demographic variable categories were significantly associated with COVID-19 knowledge (Table 1). Females were 11% more knowledgeable than males, and respondents aged 45–54 were more knowledgeable about the COVID-19 variants and vaccines (OR = 6.35, 95% CI: 2.97, 13.58). Zimbabweans and Malaysians scored worse on knowledge than the Bangladeshi respondents (OR = 0.21, 95% CI: 0.11, 0.42; OR = 0.26, 95% CI: 0.14, 0.48). Similar observations could be drawn at the country level as well. Respondents with post-graduate education were more likely to have a good Knowledge score (OR = 2.20, 95% CI: 1.53, 3.16). As expected, some high school respondents displayed less knowledge than the college graduates (OR = 0.11, 95% CI: 0.03, 0.36). However, full-time employed respondents were 42% more likely to be knowledgeable than the self-employed ones (OR = 0.58, 95% CI: 0.42, 0.79). 

In the multiple logistic regression analysis, the categories of age (45–54; AOR = 5.81, 95% CI: 2.30, 14.69)), country (USA (AOR= 3.16, 95% CI: 1.35, 7.38), and education (post-graduate school (AOR = 1.83, 95% CI: 1.23, 2.74)), with good knowledge remained highly significant (Table 1). The negative association of living in Malaysia (AOR = 0.23, 95% CI: 0.12, 0.47), having completed a trade or technical school (AOR = 0.41, 95% CI: 0.21, 0.80), and being retired (AOR = 0.45, 95% CI: 0.21, 0.96) remained significant.

### 3.5. Attitude

Table S2 provides the list of questions used to assess attitude. The majority of the study population found the vaccines to be safe and free of harmful ingredients. A neutral attitude towards the side effects was observed in 25% of the participants, whereas almost 28% were neutral about the effectiveness of the COVID-19 vaccine. Around 46% of the respondents disagreed with reported negative attitudes on social and mainstream media. As evidenced in Table 2, eight categories in the univariate model were significantly associated with a good attitude. Respondents with Zimbabwean nationality had a positive association (OR = 4.91, 95% CI: 2.13, 11.29). A highly positive association was also found between good attitude and respondents studying in high school (OR = 10.10, 95% CI: 2.35, 43.34). However, the 25–34- and 55–64-years age-group respondents were 37% and 60% less likely to have good attitude than those in the 15–24 age group (OR = 0.63, 95% CI: 0.44, 0.88; OR = 0.40, 95% CI: 0.19,0.88). In the multiple logistic regression analysis, country (USA (AOR = 0.36, 95% CI:0.18, 0.72) and education (post-graduate (AOR = 0.56, 95% CI: 0.39, 0.82) continued to have significantly negative association with good attitude level among respondents. 

### 3.6. Practice

To assess practice, the questions referenced behaviors in the past six months (see Table S3). When asked about going to crowded places, 338 respondents reported always avoiding, 376 occasionally avoiding, and 67 never avoiding these locations. In the three months before responding to the survey, 92% of the respondents did not travel internationally. Eight demographic factors were significantly related to good practice in the univariate logistic regression model (Table 3). Respondents who were in the 35–44 age group and those who were postgraduates were highly likely to have good practices (OR = 1.81, 95% CI: 1.07, 3.05; OR = 2.26, 95% CI: 1.37, 3.73). Other factors that had significant negative associations with practice included nationality ((American (OR = 0.17, 95% CI: 0.07, 0.43), being Colombian (OR = 0.13, 95% CI: 0.06, 0.29)), country (Colombia (OR = 0.15, 95% CI: 0.10, 0.25), education (trade or technical school (OR = 0.34, 95% CI: 0.20, 0.58), and employment (self-employed (OR = 0.43, 95% CI: 0.29, 0.64). 

The sex variable was not related to good practices in the multivariate model as observed in Table 3. A country such as Colombia and the USA were negatively associated with good practice. This implies that good practice levels are 85%, and 81% more likely in Bangladesh than in Colombia (AOR = 0.15, 95% CI: 0.08,0.28), or in the USA (AOR = 0.19, 95% CI: 0.09, 0.40), respectively.

## 4. Discussion

Vaccine hesitancy continues to be a public health threat, contributing to the development of variants. Research indicates that most people were infected with the UK/Alpha variant in January this year, and by March, the South African/Beta variant took the lead. The World Health Organization (WHO) noted that as of 20 July 2021, the prevalence of Delta among the specimens sequenced over the past four weeks (from 22 June 2021 to 20 July 2021) exceeded 75% in many countries worldwide including Australia, Bangladesh, Botswana, China, Denmark, India, Indonesia, Israel, Portugal, Russia, Singapore, South Africa, and the UK [24]. According to data within the last month (as of 4 July 2021), 98% of people were infected with the more virulent Delta variant (B.1.617.2) [25]. Plans to reach herd immunity during the COVID-19 pandemic remain threatened by variant development, unequal vaccine distribution, misinformation, knowledge gaps, vaccine safety concerns, and distrust, and many social, political, cultural, and personal factors (attitudes and practices) have caused growing hesitancy and anxiety surrounding COVID-19 vaccination [26,27].

Our findings show that, overall, good KAP levels are prominently higher among post-graduate and college graduate respondents and those who are in the age group of 25–34 years [28]. Previous studies have linked better education to low vaccine hesitancy and higher acceptability. Respondents in the age group of 45–54 years had almost six times higher knowledge levels than those aged between 15 and 24 years, which may be related to previous experiences and education. The odds of having good knowledge were higher in people who have completed the post-college education (graduate school). Respondents who were employed part-time were 16% less likely (OR = 0.84) to be knowledgeable than the full-time employed. Citizens’ concerns must be alleviated and evidence-based information provided to patients and healthcare providers utilizing the media outlets (e.g., social media) and resources used most often by individuals [29,30]. However, it was also observed that around 49.68% of the respondents had not yet taken the vaccine, even though almost 92% of the total number of respondents claimed that they were aware of the COVID-19 variants and seriousness associated with them. In our study, we found that approximately 53% of respondents had good knowledge of vaccines, 55% had a positive attitude, and 79% reported taking appropriate practice measures to prevent COVID-19 infection and transmission. Although the mean knowledge score among all the respondents was found to be 24, a significant proportion of the population remains hesitant to receive the COVID-19 vaccine and antivaccine movements continue to hinder efforts to achieve herd immunity. USA-based respondents are three times more likely to have good knowledge levels than those in Bangladesh (OR = 3.16, *p*-value = 0.01), which is expected per socioeconomic and educative country profiles. 

Many individuals remain doubtful as to the safety (13%) and effectiveness (27%) of COVID-19 vaccination, and without the healthcare community, negative attitudes towards the vaccine persist [31]. Studies show that only about 5% of the human populations are at risk of severe infection, requiring admission to an intensive care unit or resulting in mortality [32]. When severe COVID-19 infections are perceived to have a low risk, individuals tend to analyze their perceived risk of suffering from vaccine side effects, driving vaccine demand down [33]. Moreover, numerous wild conspiracy theories sprouted through social media [34]. There is a significant relationship between organizations on social media and public doubts of vaccine safety. In addition, there is a substantial relationship between foreign disinformation campaigns and declining vaccination coverage [35]. Understanding the threat posed by anti-vaccination efforts on social media and the impact they have on individual attitudes is critical to ensuring the success of worldwide COVID-19 vaccination programs. The need for studying country-specific causes of vaccine hesitancy is critical for improving the rate of vaccinations to achieve herd immunity [36,37].

In general, COVID-19 infection control and mitigation practices in this study were significantly better among participants from India than in Bangladesh with an AOR of 3.68. High-school graduates and post-graduates showed good practices compared to the college graduate. The highest average practice score of 8.3 was observed among those living in India and Malaysia. While our study sample had multinational strength, it was also limited to those respondents who could access the internet. Moreover, 70% of responses of our study population were between 15 and 34 years of age. One of the possible reasons could be that our survey was web-based and younger populations are considered to be internet savvy. Our findings could therefore be strengthened if similar studies were conducted in other parts of the world and methods that capture more respondents with characteristics that more closely represent those of the general populations employed. Findings from this study will also help in vaccination campaigns among ethnic minorities in both developed and developing countries [38,39].

Our study included a convenience sample of highly educated people, as 66.3% had at least a college degree. Thus, the experience of this study population may not reflect the experience of their typical country’s population. Moreover, the collection of data using web-based instruments further restricted the sample to those with access to computers and the internet. Therefore, cautious interpretations of the results are warranted. Nevertheless, specific items in the Attitude domain can help gauge vaccine hesitancy. In addition, this study involved collaborations among investigators and data collection of participants in at least six countries over four continents.

Moreover, given the high proportion of people with a college or graduate degree in the sample, it is not surprising to see that those with lower education had lower knowledge scores. However, it was surprising to see that those with a middle school or high school education were more likely to have good attitude scores and practice scores than those with a college education. Thus, it is possible to affect good attitude and good practice in those with lower formal education possibly with good social media campaigns to improve vaccine uptake and lower hesitancy. This is especially important in low-income countries where vaccines may not be widely available, and good attitude and practice are necessary to help reduce widespread infections.

## 5. Conclusions

With the emergence of the SARS-CoV-2/COVID-19 pandemic, the need for developing vaccines was not only relevant but urgent, given the spread and associated health outcomes. Access to the COVID-19 vaccines has differed in high-income countries compared to mid-and low-income countries; however, that does not appear to be the greatest modifiable and contributing factor to COVID-19 infection. Nonetheless, many individuals remain doubtful as to the safety and efficacy of COVID-19 vaccination. As with other vaccines, public acceptability is incomplete, and hesitance may interfere with vaccination plans. Education and country of residence appear to affect knowledge and influence attitude and practices. Knowledge on how diverse populations perceive the risk of COVID-19 vaccination may contribute to the development of an effective public health program. Understanding public trust and confidence during health emergencies is vital to enhance trust and promote confidence and compliance with recommendations and measures. Understanding the threat posed by anti-vaccination efforts on social media and the impact they have on individual attitudes is also critically important to ensure the success of worldwide COVID-19 vaccination programs. Without the healthcare community activists, as well as political and religious leaders speaking in one voice, significant distrust and negative attitudes towards the vaccine persist, vaccination rates remain suboptimal, COVID-19 infections spread, variants develop, and needless deaths will continue to occur. The weak positive association (r = 0.32, *p* = 0.000006) between Knowledge and Practice presents an opportunity to consider the KAP levels identified in this study in guiding public health program development, supporting the education needs of individual communities, addressing misinformation, and guiding global practice change.

## Figures and Tables

**Table 1:** Univariate and multiple logistic regression analysis showing predictors of knowledge levels (good vs. poor) (*n* = 781).

Independent Variable		Respondents with Score > 23.5		Univariate Logistic Regression				Multiple Logistic Regression			
		*n*	%	OR	*p*-Value	95% CI		AOR	*p*-Value	95% CI	
Sex											
	Male ^®^	206	51.76								
	Female	208	54.31	1.11	0.48	0.84	1.47	1.28	0.14	0.92	1.77
Age groups											
	15–24 ^®^	104	44.07								
	25–34	177	56.55	1.65 *	0.00	1.18	2.32	1.10	0.72	0.66	1.84
	35–44	64	46.38	1.10	0.67	0.72	1.67	0.92	0.80	0.49	1.75
	45–54	45	83.33	6.35 *	0.00	2.97	13.58	5.81 *	0.00	2.30	14.69
	55–64	17	56.67	1.66	0.20	0.77	3.57	2.43	0.12	0.80	7.38
	65–74	7	70.00	2.96	0.12	0.75	11.73	7.47	0.07	0.86	64.82
Nationality											
	Bangladeshi ^®^	173	62.45								
	American	25	75.76	1.88	0.14	0.82	4.32				
	Colombian	101	42.62	0.45 *	0.00	0.31	0.64				
	Indian	66	65.35	1.13	0.61	0.70	1.83				
	Malaysian	18	30.51	0.26 *	0.00	0.14	0.48				
	Zimbabwean	13	26.00	0.21 *	0.00	0.11	0.42				
	Other	18	75.00	1.80	0.23	0.69	4.69				
Country											
	Bangladesh ^®^	164	61.19								
	Colombia	102	43.40	0.49 *	0.00	0.34	0.69	0.56 *	0.02	0.34	0.92
	India	61	63.54	1.11	0.69	0.68	1.79	1.37	0.29	0.77	2.44
	Malaysia	18	31.03	0.29 *	0.00	0.16	0.52	0.23 *	0.00	0.12	0.47
	USA	42	84.00	3.33 *	0.00	1.50	7.37	3.16 *	0.01	1.35	7.38
	Zimbabwe	14	26.42	0.23 *	0.00	0.12	0.44	0.60	0.27	0.25	1.47
	Other	13	61.90	1.03	0.95	0.41	2.57	1.33	0.59	0.48	3.71
Education											
	College graduate ^®^	141	52.03								
	Grade school	6	37.50	0.55	0.26	0.20	1.56	0.39	0.12	0.12	1.29
	Junior high or middle school	7	41.18	0.65	0.39	0.24	1.75	0.77	0.66	0.25	2.38
	Some high school	3	10.34	0.11 *	0.00	0.03	0.36	0.13*	0.01	0.03	0.59
	High school graduate	52	52.00	1.00	1.00	0.63	1.58	1.25	0.47	0.68	2.28
	Trade or technical school	19	25.33	0.31 *	0.00	0.18	0.55	0.41 *	0.01	0.21	0.80
	Some college	12	46.15	0.79	0.57	0.35	1.77	0.46	0.10	0.18	1.16
	Post college (graduate school)	174	70.45	2.20 *	0.00	1.53	3.16	1.83 *	0.00	1.23	2.74
Employment											
	Employed full time ^®^	221	60.22								
	Employed part time	19	55.88	0.84	0.62	0.41	1.70	0.91	0.81	0.41	2.01
	Retired	7	43.75	0.47 *	0.03	0.24	0.95	0.45 *	0.04	0.21	0.96
	Self-employed	22	44.90	0.58 *	0.00	0.42	0.79	1.11	0.71	0.65	1.89
	Student	126	46.67	0.51	0.20	0.19	1.41	0.30	0.17	0.05	1.64
	Unemployed	15	41.67	0.54 *	0.04	0.30	0.98	0.53	0.07	0.26	1.05
	Other	4	44.44	0.53	0.35	0.14	2.00	0.22 *	0.04	0.05	0.96

OR: Odds Ratio by univariate analysis; AOR: Adjusted Odds Ratio by multiple logistic regression (variables in the model: Sex, Age group, Country, Education, Employment). 95% CI: Confidence interval at the 95% level. *: *p*-value ≤ 0.05 considered to be significant; ^®^: Reference variable.

**Table 2:** Univariate and multiple logistic regression analysis showing predictors of attitude levels (Good vs. Poor) (*n* = 781).

Independent Variable		Respondents with a Score >27.5		Univariate Logistic Regression				Multiple Logistic Regression			
		*n*	%	OR	*p*-Value	95% CI		AOR	*p*-Value	95% CI	
Sex											
	Male ^®^	229	57.54								
	Female	203	53.00	0.83	0.20	0.63	1.10	0.81	0.20	0.60	1.11
Age groups											
	15–24 ^®^	147	62.29								
	25–34	159	50.80	0.63 *	0.01	0.44	0.88	0.78	0.32	0.47	1.28
	35–44	79	57.25	0.81	0.34	0.53	1.24	0.98	0.94	0.53	1.81
	45–54	28	51.85	0.65	0.16	0.36	1.18	0.90	0.78	0.42	1.92
	55–64	12	40.00	0.40 *	0.02	0.19	0.88	0.47	0.15	0.16	1.33
	65–74	7	70.00	1.41	0.62	0.36	5.60	1.89	0.49	0.31	11.40
Nationality											
	Bangladeshi ^®^	154	55.60								
	American	9	27.27	0.30 *	0.00	0.13	0.67				
	Colombian	129	54.43	0.95	0.79	0.67	1.35				
	Indian	53	52.48	0.88	0.59	0.56	1.39				
	Malaysian	35	59.32	1.16	0.60	0.66	2.06				
	Zimbabwean	43	86.00	4.91 *	0.00	2.13	11.29				
	Other	9	37.50	0.48	0.09	0.20	1.13				
Country											
	Bangladesh ^®^	149	55.60								
	Colombia	129	54.89	0.97	0.87	0.68	1.38	0.72	0.18	0.44	1.17
	India	52	54.17	0.94	0.81	0.59	1.51	0.81	0.46	0.47	1.41
	Malaysia	34	58.62	1.13	0.67	0.64	2.01	0.98	0.95	0.52	1.84
	USA	16	32.00	0.38 *	0.00	0.20	0.71	0.36*	0.00	0.18	0.72
	Zimbabwe	45	84.91	4.49 *	0.00	2.04	9.90	1.80	0.25	0.67	4.88
	Other	7	33.33	0.40	0.06	0.16	1.02	0.37	0.06	0.14	1.02
Education											
	College graduate ^®^	155	57.20								
	Grade school	9	56.25	0.96	0.94	0.35	2.66	0.85	0.77	0.29	2.49
	Junior high or middle school	14	82.35	3.49 *	0.05	0.98	12.44	3.52	0.07	0.91	13.56
	Some high school	27	93.10	10.10 *	0.00	2.35	43.34	5.37	0.06	0.93	31.15
	High school graduate	51	51.00	0.78	0.29	0.49	1.23	0.84	0.56	0.47	1.51
	Trade or technical school	50	66.67	1.50	0.14	0.87	2.56	1.85	0.06	0.99	3.46
	Some college	18	69.23	1.68	0.24	0.71	4.01	1.89	0.18	0.75	4.75
	Post college (graduate school)	108	43.72	0.58 *	0.00	0.41	0.82	0.56 *	0.00	0.39	0.82
Employment											
	Employed full time ^®^	192	52.32								
	Employed part time	20	58.82	1.30	0.47	0.64	2.66	1.24	0.58	0.58	2.66
	Retired	10	62.50	1.14	0.71	0.57	2.27	1.05	0.90	0.50	2.20
	Self-employed	31	63.27	1.23	0.20	0.90	1.69	0.78	0.34	0.47	1.30
	Student	155	57.41	1.52	0.43	0.54	4.27	1.39	0.65	0.33	5.92
	Unemployed	20	55.56	1.57	0.15	0.85	2.91	1.62	0.15	0.84	3.15
	Other	4	44.44	0.73	0.64	0.19	2.76	0.79	0.75	0.18	3.39

OR: Odds Ratio by univariate analysis; AOR: Adjusted Odds Ratio by multiple logistic regression (variables in the model: Sex, Age group, Country, Education, Employment). 95% CI: Confidence interval at the 95% level. *: *p*-value ≤ 0.05 considered to be significant; ^®^: Reference variable.

**Table 3:** Univariate and multiple logistic regression analysis showing predictors of practice levels (Good vs. Poor) (*n* = 781).

Independent Variable		Respondents with a Score > 5.5		Univariate Logistic Regression				Multiple Logistic Regression			
		*n*	%	OR	*p*-Value	95% CI		AOR	*p*-Value	95% CI	
Sex											
	Male ^®^	320	80.40								
	Female	300	78.33	0.88	0.47	0.62	1.25	1.24	0.29	0.83	1.85
Age groups											
	15–24 ^®^	171	72.46								
	25–34	256	81.79	1.71 *	0.01	1.14	2.56	1.23	0.50	0.67	2.25
	35–44	114	82.61	1.81 *	0.03	1.07	3.05	1.86	0.11	0.87	3.97
	45–54	45	83.33	1.90	0.10	0.88	4.11	1.84	0.22	0.70	4.89
	55–64	25	83.33	1.90	0.21	0.70	5.18	3.79	0.07	0.90	15.92
	65–74	9	90.00	3.42	0.25	0.42	27.54	34.12 *	0.02	1.80	647.89
Nationality											
	Bangladeshi ^®^	250	90.25								
	American	18	54.55	0.13 *	0.00	0.06	0.29				
	Colombian	139	58.65	0.15 *	0.00	0.10	0.25				
	Indian	97	96.04	2.62	0.08	0.89	7.68				
	Malaysian	53	89.83	0.95	0.92	0.38	2.43				
	Zimbabwean	42	84.00	0.57	0.19	0.24	1.33				
	Other	21	87.50	0.76	0.67	0.21	2.70				
Country											
	Bangladesh ^®^	242	90.30								
	Colombia	137	58.30	0.15 *	0.00	0.09	0.24	0.15 *	0.00	0.08	0.28
	India	92	95.83	2.47	0.10	0.84	7.27	3.68 *	0.03	1.15	11.74
	Malaysia	52	89.66	0.93	0.88	0.36	2.38	0.92	0.87	0.33	2.52
	USA	34	68.00	0.23 *	0.00	0.11	0.47	0.19 *	0.00	0.09	0.40
	Zimbabwe	44	83.02	0.53	0.13	0.23	1.20	0.55	0.32	0.17	1.80
	Other	19	90.48	1.02	0.98	0.22	4.63	1.01	0.99	0.21	4.84
Education											
	College graduate ^®^	214	78.97								
	Grade school	15	93.75	4.00	0.18	0.52	30.89	3.39	0.26	0.40	28.39
	Junior high or middle school	12	70.59	0.64	0.42	0.22	1.89	1.48	0.54	0.42	5.27
	Some high school	24	82.76	1.28	0.63	0.47	3.50	1.78	0.47	0.38	8.38
	High school graduate	70	70.00	0.62	0.07	0.37	1.04	2.16 *	0.03	1.07	4.38
	Trade or technical school	42	56.00	0.34 *	0.00	0.20	0.58	1.15	0.68	0.59	2.25
	Some college	22	84.62	1.46	0.50	0.49	4.42	0.91	0.89	0.26	3.24
	Post college (graduate school)	221	89.47	2.26 *	0.00	1.37	3.73	1.97 *	0.02	1.11	3.47
Employment											
	Employed full time ^®^	313	85.29								
	Employed part time	26	76.47	0.56	0.18	0.24	1.30	0.89	0.81	0.34	2.31
	Retired	12	75.00	0.71	0.45	0.30	1.71	0.79	0.65	0.29	2.16
	Self-employed	40	81.63	0.43 *	0.00	0.29	0.64	0.88	0.69	0.47	1.63
	Student	193	71.48	0.52	0.27	0.16	1.66	0.13 *	0.04	0.02	0.94
	Unemployed	29	80.56	0.77	0.50	0.35	1.67	1.13	0.78	0.48	2.68
	Other	7	77.78	0.60	0.54	0.12	2.98	0.32	0.20	0.06	1.82

OR: Odds Ratio by univariate analysis; AOR: Adjusted Odds Ratio by multiple logistic regression (variables in the model: Sex, Age group, Country, Education, Employment). 95% CI: Confidence interval at the 95% level. *: *p*-value ≤ 0.05 considered to be significant; ^®^: Reference variable.

## Data Availability

Data will be shared based upon request through the corresponding author.

## Supplementary Materials

The following are available online at https://www.mdpi.com/article/10.3390/bs11110148/s1, Table S1a. Descriptive summary of COVID-19 variants knowledge, Table S1b. Descriptive summary of COVID-19 vaccine knowledge, Table S2. Descriptive summary of attitude towards COVID-19 vaccine, Table S3. Descriptive summary of practices towards COVID-19, Table S4. Scoring system for the questionnaire on knowledge, attitude, and practices related to COVID-19.
