# Supplementary material for: COVID-19 Vaccine Hesitancy and Emerging Variants: Evidence from Six Countries
Source: Behav Sci (Basel). 2021 Oct 28;11(11):148. doi: 10.3390/bs11110148 (PMC8614952; doi:10.3390/bs11110148)
Supplement: Supplementary file 1 [file behavsci-11-00148-s001.zip › behavsci-1401399-SI.pdf]

Supplement tables

Table S1(a). Descriptive summary of COVID-19 variants knowledge.

Knowledge Questions on COVID-19 Variants (n=781)	Yes		No		Do not know /Not sure	
	n	%	n	%	n	%
• Are you aware that there are new variants of COVID-19?	722	92.45	59	7.55	-	-
• How many current strains of COVID-19 are you aware of?						
1	60	7.68	-	-	-	-
2	185	23.69	-	-	-	-
3	215	27.53	-	-	-	-
More than 3	321	41.10	-	-	-	-
• Which among the following are the new variants of COVID-19? (Select all that apply)					542	69.40
One correct	117	14.98	-	-		
Two correct	48	6.15	-	-		
Three correct	47	6.02	-	-		
All correct	27	3.46	-	-		
• What countries are the new variants from? (Select all that apply)					108	13.83
One correct	292	37.39	-	-		
Two correct	152	19.46	-	-		
Three correct	155	19.85	-	-		
Four correct	57	7.30	-	-		
All correct	17	2.18	-	-		
• Do you think the new variants spread faster/more contagious?	631	80.79	28	3.59	122	15.62
• Will these new variants cause different symptoms?	431	55.19	156	19.97	194	24.84
• What symptoms do you expect to notice with the new COVID-19 variants? (Select all that apply)						
Cough	559	71.57	-	-	-	-
Fatigue/weakness	566	72.47	-	-	-	-
Headache	547	70.04	-	-	-	-
Muscle aches	523	66.97	-	-	-	-
Sore throat	502	64.28	-	-	-	-
Fever	603	77.21	-	-	-	-
Loss of taste	514	65.81	-	-	-	-
Loss of smell	534	68.37	-	-	-	-
Other (Abdominal pain, Breathlessness, Vomiting, Diarrhoea)	23	2.94	-	-	-	-

• What measures are necessary to prevent the spread of new variants? (Select all that apply)					24	3.07
One correct	43	5.51	-	-		
Two correct	26	3.33	-	-		
Three correct	100	12.80	-	-		
Four correct	201	25.74	-	-		
All correct	387	49.55	-	-		
• Do you think current diagnostic tests are able to detect the new variants?	448	57.36	115	14.72	218	27.91
• What is your source of information on new strains of COVID-19? (Select all that apply)						
Radio	101	12.93	-	-	-	-
TV	420	53.78	-	-	-	-
Newspaper	242	30.99	-	-	-	-
Social media (Twitter, Facebook, Instagram, YouTube etc.)	516	66.07	-	-	-	-
Internet (WHO, FDA, CDC websites)	456	58.39	-	-	-	-
Relatives and friends	187	23.94	-	-	-	-

Table S1(b). Descriptive summary of COVID-19 vaccine knowledge.

Knowledge about COVID-19 Vaccine	Yes		No		Not taken	
	n	%	n	%	n	%
• Have you received the COVID-19 vaccine yet?	393	50.32			388	49.68
• If yes, how many doses of the COVID-19 vaccine did you receive?						
One	77	19.59	-	-	-	-
Two	316	80.41	-	-	-	-
• Which vaccine did you receive?						
Pfizer	160	40.71	-	-	-	-
Moderna	16	4.07	-	-	-	-
Johnson & Johnson	5	1.27	-	-	-	-
Sinopharm	7	1.78	-	-	-	-
Sinovac	12	3.05	-	-	-	-
Covaxin	6	1.53	-	-	-	-
Covishield	185	47.07	-	-	-	-
Other	2	0.51	-	-	-	-
• Have you experienced any side effects after taking a vaccine shot?	242	61.58	151	38.42	-	-

• If yes, what are the side effects experienced?						
Vaccination site discomfort (pain/redness/swelling)	160	66.12	-	-	-	-
Tiredness	112	46.28	-	-	-	-
Headache	97	40.08	-	-	-	-
Muscle pain	121	50.00	-	-	-	-
Chills	44	18.18	-	-	-	-
Fever	102	42.15	-	-	-	-
Nausea	13	5.37	-	-	-	-
Other	0	0.00	-	-	-	-
• Did you get a COVID-19 infection after getting vaccinated?	70	17.81	52	13.23	-	-
• Did anyone in your family/friends get a COVID-19 infection after getting vaccinated?	20	2.56	368	47.12	-	-

Table S2. Descriptive summary of attitude towards COVID-19 vaccine

Attitude towards COVID-19 Vaccines	Completely disagree		Somewhat disagree		Neutral		Somewhat agree		Completely agree	
	n	%	n	%	n	%	n	%	n	%
The COVID-19 vaccines are safe.	39	4.99	70	8.96	203	25.99	192	24.58	277	35.47
The COVID-19 vaccines contain dangerous ingredients.	343	43.92	145	18.57	216	27.66	49	6.27	28	3.59
Worried about the serious side effects from the COVID-19 vaccine.	237	30.35	130	16.65	197	25.22	152	19.46	65	8.32
Don't have enough information about the COVID-19 vaccine to decide whether to get it myself.	333	42.64	118	15.11	167	21.38	89	11.40	74	9.48
Waiting to get the vaccine because it's new.	245	31.37	106	13.57	193	24.71	123	15.75	114	14.60
The COVID-19 vaccines are effective at preventing COVID-19 infection.	106	13.57	110	14.08	217	27.78	202	25.86	146	18.69
There are more effective ways to prevent COVID-19 than a vaccine.	201	25.74	160	20.49	230	29.45	111	14.21	79	10.12
Contracting COVID-19 provides better immunity than a vaccine.	216	27.66	150	19.21	244	31.24	109	13.96	62	7.94
Reports on social media have made to reconsider choice to receive the COVID-19 vaccine.	268	34.31	94	12.04	214	27.40	119	15.24	86	11.01
Read reports in the mainstream media that have made to reconsider choice to receive the COVID-19 vaccine.	279	35.72	85	10.88	214	27.40	124	15.88	79	10.12

Religious leaders have advocated against COVID-19 vaccination.	428	54.80	66	8.45	210	26.89	40	5.12	37	4.74

Table S3. Descriptive summary of practices towards COVID-19.

Practices related to COVID-19	Always/Yes /Very comfortable		Occasionally/ No/ Maybe/Comfortable		Never/No/ Neutral		Uncomfortable		Very uncomfortable	
	n	%	n	%	n	%	n	%	n	%
Avoided touching your nose, mouth, and eyes with your hands unwashed in the last 3 months	261	33.42	423	54.16	97	12.42	-	-	-	-
Avoided going to any crowded places in last 3 months	338	43.28	376	48.14	67	8.58	-	-	-	-
Travelled outside your country in last 3 months	53	6.79	723	92.57	5	0.64	-	-	-	-
Willing to avoid unnecessary travel (Domestic/International) due to the rapid spread of new variants	569	72.86	116	14.85	96	12.29	-	-	-	-
Shared knowledge on new variants with family/friends/peers	333	42.64	298	38.16	150	19.21	-	-	-	-
Comfortable in sharing your knowledge on COVID-19 variants with your family/friends/peer	239	30.60	257	32.91	221	28.30	61	7.81	3	0.38

Appendix

COVID-19 Vaccine Hesitancy and Emerging Variants: Evidence from Six Countries

DEMOGRAPHICS:

1. Your sex (*check one*): ☐ Male ☐ Female

2. Your age (*in years*): _________

3. What is your nationality? _________________ (*Please specify*)

4. Which country do you live in? _________________ (*Please specify*)

5. Which state do you live in? _________________ (*Please specify*)

6. What is the highest level of education that you have completed?
   - ☐ Did not complete grade school
   - ☐ Grade school
   - ☐ Junior high or middle school
   - ☐ Some high school
   - ☐ High school graduate
   - ☐ Trade or technical school
   - ☐ Some college
   - ☐ College graduate
   - ☐ Post-college (graduate school)

7. What is your current employment status?
   - ☐ Employed full time
   - ☐ Employed part-time
   - ☐ Unemployed (currently looking for work)
   - ☐ Unemployed (currently not looking for work)
   - ☐ Student
   - ☐ Home-maker
   - ☐ Retired
   - ☐ Self-employed
   - ☐ Unable to work
   - ☐ Other _______________ (*Please specify)*

8. What is your current occupation? _________________ (*Please specify*)

KNOWLEDGE

1. Are you aware that there are new variants of COVID-19?
   - O Yes (If yes move to next question)
   - O No
2. How many current strains of COVID-19 are you aware of?
   - O 1
   - O 2

- 3
- More than 3

3. Which among the following are the new variants of COVID-19? (Select all that apply)
   - B.1.1.7 lineage
   - B.1.351 lineage
   - P.1 lineage
   - B.1.617 lineage
   - Don’t know/Not sure
4. What countries are the new variants from? (Select all that apply)
   - UK
   - South Africa
   - Brazil
   - US
   - India
   - Don’t know/Not sure
5. Do you think the new variants spread faster/more contagious?
   - Yes
   - No
   - Don’t know/Not sure
6. Will these new variants cause different symptoms?
   - Yes
   - No
   - Don’t know/Not sure
7. What symptoms do you expect to notice with the new COVID-19 variants? (Select all that apply)
   - Cough
   - Fatigue/weakness
   - Headache
   - Muscle aches
   - Sore throat
   - Fever
   - Loss of taste
   - Loss of smell
   - Other_________
8. What measures are necessary to prevent the spread of new variants? (Select all that apply)
   - Masking/Double masking
   - Washing hands frequently

- Ventilating indoor spaces
- Staying 6feet apart from others
- Avoiding crowds
- Don’t know/Not sure

9. Do you think current diagnostic tests can detect the new variants?
   - Yes
   - No
   - Don’t know/Not sure
10. What is your source of information on new strains of COVID-19? (Select all that apply)
    - Radio
    - TV
    - Newspaper
    - Social media (Twitter, Facebook, Instagram, YouTube, etc.)
    - Internet (WHO, FDA, CDC websites)
    - Relatives and friends
    - Others ___________ (please specify)

Vaccination status

1. Have you received the COVID-19 vaccine yet?
   - Yes
   - No

If yes,

2. How many doses of the COVID-19 vaccine did you receive?
   - One
   - Two

3. Which vaccine did you receive?
   - Pfizer
   - Moderna
   - Johnson & Johnson
   - Sinopharm
   - Sinovac
   - Covaxin

- Covishield
- Others ___________ (please specify)

4. Have you experienced any side effects after taking a vaccine shot?
   - Yes
   - No

   If yes….

5. What are the side effects experienced?
   - Vaccination site discomfort (pain/redness/swelling)
   - Tiredness
   - Headache
   - Muscle pain
   - Chills
   - Fever
   - Nausea
   - Others _________________ (please specify)
6. Did you get a COVID-19 infection after getting vaccinated?
   - Yes
   - No
7. Did anyone in your family/friends get a COVID-19 infection after getting vaccinated?
   - Yes
   - No

ATTITUDES & BELIEF

**Perceived Safety**	Completely Disagree	Somewhat Disagree	Neutral	Somewhat Agree	Completely Agree
The COVID-19 vaccines are safe.	☐	☐	☐	☐	☐

The COVID-19 vaccines contain dangerous ingredients.	☐	☐	☐	☐	☐
I am worried about the serious side effects of the COVID-19 vaccine.	☐	☐	☐	☐	☐
I don't have enough information about the COVID-19 vaccine to decide whether to get it myself.	☐	☐	☐	☐	☐
The COVID-19 vaccine is so new that I want to wait a while before deciding if I should get it.	☐	☐	☐	☐	☐

**Perceived Effectiveness**	Completely Disagree	Somewhat Disagree	Neutral	Somewhat Agree	Completely Agree
The COVID-19 vaccines are effective at preventing COVID-19 infection.	☐	☐	☐	☐	☐
There are more effective ways to prevent COVID-19 than a vaccine.	☐	☐	☐	☐	☐
Contracting COVID-19 provides better immunity than a vaccine.	☐	☐	☐	☐	☐

**Community Involvement**	Completely Disagree	Somewhat Disagree	Neutral	Somewhat Agree	Completely Agree
I have heard/read reports on social media that have made me reconsider my choice to receive the COVID-19 vaccine.	☐	☐	☐	☐	☐
I have heard/read reports in the mainstream media that have made me reconsider my choice to receive the COVID-19 vaccine.	☐	☐	☐	☐	☐
My religious leaders have advocated against COVID-19 vaccination.	☐	☐	☐	☐	☐

PRACTICE

1. In the past 3 months, have you avoided touching your nose, mouth, and eyes with your hands unwashed?
   - O Always
   - O Occasionally

- Never

2. In the past 3 months, have you avoided going to any crowded places?
   - Always
   - Occasionally
   - Never
3. In the past 3 months, have you traveled outside your country?
   - Yes
   - No
   - Prefer not to disclose
4. Due to the rapid spread of new variants, are you willing to avoid unnecessary travel (Domestic/International)?
   - Always
   - Occasionally
   - Never
5. In recent days, have you shared your knowledge on new variants with family/friends/peers?
   - Always
   - Occasionally
   - Never
6. How comfortable are you in sharing your knowledge on COVID-19 variants with your family/friends/peers?
   - Very comfortable
   - Comfortable
   - Neutral
   - Uncomfortable
   - Very uncomfortable

Thank you for your kind participation in this survey.
